# CEP295 interacts with microtubules and is required for centriole elongation

**DOI:** 10.1242/jcs.186338

**Published:** 2016-07-01

**Authors:** Ching-Wen Chang, Wen-Bin Hsu, Jhih-Jie Tsai, Chieh-Ju C. Tang, Tang K. Tang

**Affiliations:** Institute of Biomedical Sciences, Academia Sinica, Taipei 11529, Taiwan

**Keywords:** Centriole assembly, Centriole duplication, Procentriole formation, Centrosome, Acetylation, Polyglutamylation

## Abstract

Centriole duplication is a tightly ordered process during which procentrioles are assembled in G1-S and elongate during S and G2. Here, we show that human CEP295 (*Drosophila* Ana1) is not essential for initial cartwheel assembly, but is required to build distal half centrioles during S and G2. Using super-resolution and immunogold electron microscopy, we demonstrate that CEP295 is recruited to the proximal end of procentrioles in early S phase, when it is also localized at the centriolar microtubule wall that surrounds the human SAS6 cartwheel hub. Interestingly, depletion of CEP295 not only inhibits the recruitments of POC5 and POC1B to the distal half centrioles in G2, resulting in shorter centrioles, it also blocks the post-translational modification of centriolar microtubules (e.g. acetylation and glutamylation). Importantly, our results indicate that CEP295 directly interacts with microtubules, and that excess CEP295 could induce the assembly of overly long centrioles. Furthermore, exogenous expression of the N-terminal domain of CEP295 exerts a dominant-negative effect on centriole elongation. Collectively, these findings suggest that CEP295 is essential for building the distal half centrioles and for post-translational modification of centriolar microtubules.

## Abstract (precis)

**Highlighted Article:** Human CEP295 interacts with microtubules and is essential for building the distal half centrioles and post-translational modification of centriolar microtubules.

## INTRODUCTION

Centrioles are microtubule-based cylinders that form centrosomes, which are the major microtubule organizing centers of animal cells, and are vital for many cellular and developmental processes. Centriole duplication comprises three major steps; initiation, elongation, and maturation (Azimzadeh and Marshall, 2010; Jana et al., 2014). Recent studies have identified a number of conserved proteins, e.g. PLK4, human SAS6 (also known as SASS6 and hereafter referred to as hSAS6), STIL, CEP135, CPAP (also known as CENPJ) and CEP120, which are essential for cartwheel formation and procentriole assembly during the early stage of centriole duplication in mammalian cells (Comartin et al., 2013; Leidel et al., 2005; Lin et al., 2013a,b; Kleylein-Sohn et al., 2007; Kohlmaier et al., 2009; Schmidt et al., 2009; Tang et al., 2009, 2011). However, we do not yet understand the molecular mechanism through which a full-length centriole is generated, nor do we know the hierarchical order of the proteins that participate in the later stages of centriole elongation.

The *Drosophila* protein, Ana1, is reportedly required for centriole duplication (Blachon et al., 2009; Dobbelaere et al., 2008; Goshima et al., 2007). Its possible human homolog, CEP295 (also known as KIAA1731), shares 15% amino acid identity (27% similarity) throughout the primary sequence (Knorz et al., 2010), and was recently reported to be required for centriole-to-centrosome conversion in mitosis (Fu et al., 2016; Izquierdo et al., 2014). However, the role of CEP295 in centriole duplication remains obscure. Here, we report a newly identified role for CEP295 in centriole duplication, and offer a mechanistic basis for this effect. We demonstrate that CEP295 directly interacts with microtubules and that excess CEP295 could induce overly long centrioles. Furthermore, we show that CEP295 is essential for building the distal half centrioles and required for post-translational modification of centriolar microtubules during centriole assembly.

## RESULTS

### Depletion of CEP295 produces shorter centrioles

**Fig. 1.**
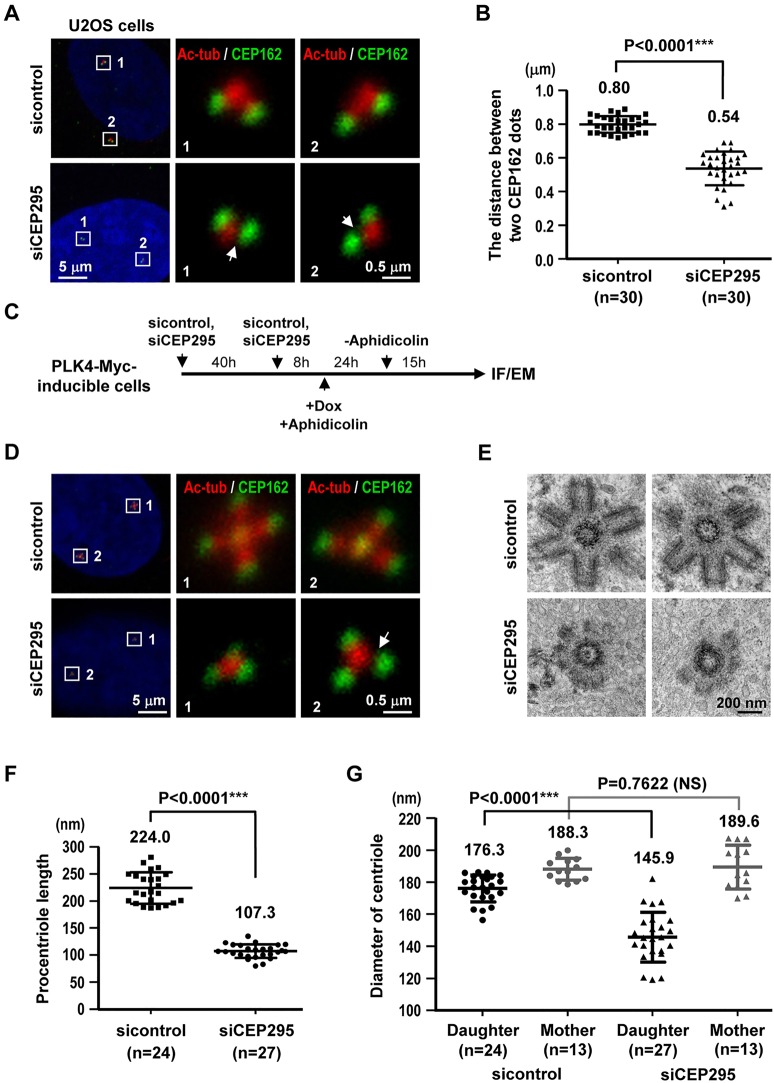
**Centriole elongation is impaired in the absence of CEP295.** (A) U2OS cells were transfected with control or CEP295-targeting siRNAs for 4 days. On day 3, aphidicolin was added to arrest cells at early S phase. After 24 h, the cells were released in fresh medium for another 8 h to enrich for G2-phase cells. The treated cells were fixed and stained with antibodies against CEP162 (green) and acetylated tubulin (Ac-tub, red). DNA was counterstained with DAPI. (B) Elongation of centrioles was calculated by measuring the distances between the centers of the two dots (representing staining for CEP162) within each pair of orthogonally arranged G2 centrioles. Error bars indicate the s.d. (standard deviation); ***P<0.0001 (two-tailed unpaired *t*-test). (C–G) PLK4-inducible cells were treated with sicontrol or siCEP295 as described in (C). IF, immunofluorescence; EM, electron microscopy; Dox, doxycycline. Cells were analyzed by immunofluorescence microscopy using the indicated antibodies (D), or by electron microscopy (E). Quantitative analysis of the centriole lengths (F) or the diameters (G) of daughter and mother centrioles acquired from electron microscopy images of sicontrol- and siCEP295-treated PLK4-inducible cells. Error bars represent the mean±s.d.; NS, not significant (two-tailed unpaired *t*-test). Arrows indicate reduced intensity of Ac-tub at the nascent centrioles.

CEP295 is reportedly essential for centriole formation (Knorz et al., 2010) and for the centriole-to-centrosome conversion (Fu et al., 2016; Izquierdo et al., 2014). Unexpectedly, however, the loss of CEP295 seemed to have no effect on initial cartwheel assembly in interphase cells (Izquierdo et al., 2014). Consistent with this finding, our results showed that CEP295 depletion did not inhibit the early recruitment of centriolar proteins (e.g. hSAS6, STIL, CPAP, CP110 and centrin) to new-born procentrioles in U2OS cells in early S phase (data not shown). Thus, we examined whether the loss of CEP295 might affect centriole elongation during late S/G2 phase. CEP295-depleted (siCEP295) or control (sicontrol) U2OS cells were synchronized at G2 phase (when the duplicated centrosomes could be clearly visualized as separate from each other) and immunostained with antibodies against acetylated-tubulin (Ac-tub, a stabilized centriole marker; Piperno et al., 1987) and CEP162 (a known centriole distal-end marker; Wang et al., 2013). Our results revealed that depletion of CEP295 did not affect the localization or intensity of CEP162 at the centrioles during G2 (Fig. 1A) (Izquierdo et al., 2014), whereas the intensity of Ac-tub at the nascent centrioles was substantially reduced (Fig. 1A, arrow). We then measured the distance between two CEP162 dots located at the distal ends of paired centrioles (one on the mother centriole, the other on the procentriole) in G2-stage cells using the method described by Azimzadeh et al. (2009). The mean distance between the two CEP162 dots in CEP295-depleted cells (mean±s.d.; 0.54±0.10 μm) was significantly less than that of control cells (0.80±0.05 μm), indicating that the procentriole length was reduced in CEP295-depleted cells (Fig. 1B). Consistent with this finding, CEP295 depletion also produced multiple shortened centrioles in PLK4-inducible cells, which were treated as described in Fig. 1C and examined by immunofluorescence staining (Fig. 1D) and electron microscopy (Fig. 1E). Further electron microscopy analysis showed that the average length of a procentriole in sicontrol cells was 224.0 nm, whereas that in CEP295-depleted cells was 107.3 nm (Fig. 1F). The diameter of the mother centriole was not significantly different between sicontrol cells (∼188.3 nm) and CEP295-depleted cells (∼189.6 nm), whereas the diameter of the procentriole (daughter) was much smaller in CEP295-depleted cells (∼145.9 nm) compared with control cells (∼176.3 nm) (Fig. 1G). We also generated U2OS-derived cell lines expressing a full-length siRNA-resistant GFP–CEP295R construct (hereafter referred to as G-CEP295R) under the control of doxycycline (Dox). Our results showed that the phenotype of shorter centrioles in CEP295-depleted cells could be effectively rescued by exogenous expression of a G-CEP295R construct (Fig. S1). Together, our results suggest that CEP295 is not essential for initial cartwheel assembly during procentriole formation, but that its depletion could produce shortened centrioles with reduced diameters.

### CEP295 is located at the microtubule wall and enriched at the proximal ends of centrioles

**Fig. 2.**
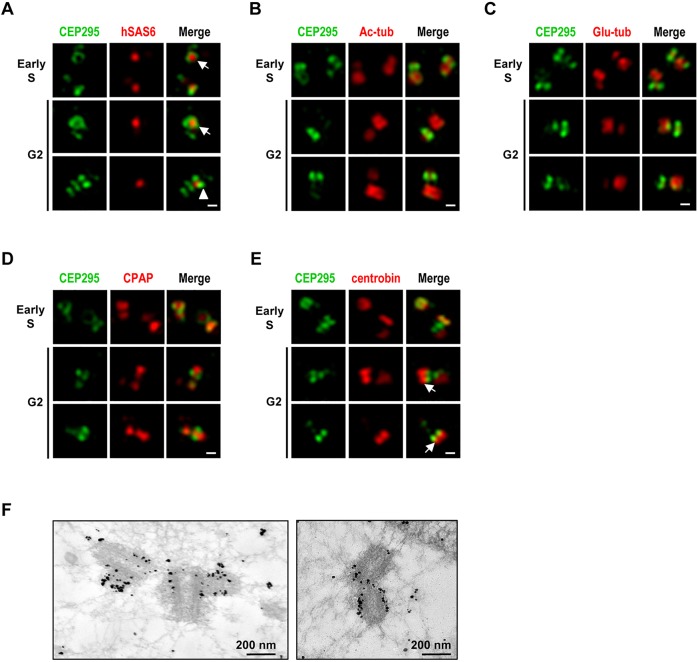
**Super-resolution (3D-SIM) analysis of the subcellular localizations of CEP295 and other centriolar proteins during centriole biogenesis.** U2OS cells were synchronized by aphidicolin for 24 h (to arrest cells at early S phase) and the synchronized cells were released in fresh medium for another 8 h (to allow progression to G2 phase). The cells were then fixed and dual stained with antibodies against CEP295 (A–E) and hSAS6 (A), Ac-tub (B), glutamylated tubulin (Glu-tub) (C), CPAP (D) or centrobin (E). Super-resolution images were acquired using a Zeiss ELYRA system. (A) Arrows indicate that CEP295 embraces hSAS6 at the proximal end of the procentriole, arrowhead indicates two-dot shape with hSAS6 in the center. (E) Arrows indicate localization of centrobin distal to CEP295 on growing centrioles. Scale bars: 0.2 μm. (F) Immunogold electron microscopy analysis of CEP295 localization.

To finely map the localization of CEP295 and examine its spatial correlation with other centriolar proteins during centriole elongation, we performed three-dimensional structured illumination microscopy (3D-SIM) and immunogold electron microscopy. U2OS cells were synchronized at early S or G2 phase and analyzed by 3D-SIM using the indicated antibodies. The top-down views of early S- or G2-centrioles showed CEP295 forming a ring-like pattern that embraces the cartwheel protein, hSAS6, at the proximal end of the procentriole (Fig. 2A, arrow). Meanwhile, side-view images of CEP295 often revealed a two-dot shape with hSAS6 in the center (Fig. 2A, arrowhead). Further immunofluorescence analyses revealed that the CEP295 signals were concentrated in regions surrounding the centriolar cylinder, as assessed by labeling with Ac-tub (Fig. 2B), glutamylated tubulin (Glu-tub; Fig. 2C), and CPAP (an inner centriolar lumen marker; Kleylein-Sohn et al., 2007) (Fig. 2D). Consistent with our findings, Fu et al. (2016) also reported that CEP295 closely surrounds the proximal part of the microtubule wall. Interestingly, centrobin, a known daughter-centriole-enriched protein (Zou et al., 2005) was localized distal to CEP295 on growing centrioles (Fig. 2E, arrow). Notably, our immunogold electron microscopy analysis directly demonstrated that the CEP295-specific gold particles clustered largely at the proximal end of centriolar microtubule wall (Fig. 2F). Collectively, our results indicate that CEP295 is localized at the centriolar microtubule wall and is especially enriched at the proximal ends of the centrioles.

### Depletion of CEP295 inhibits the CPAP- or CEP120-overexpression-induced extension of centrioles

**Fig. 3.**
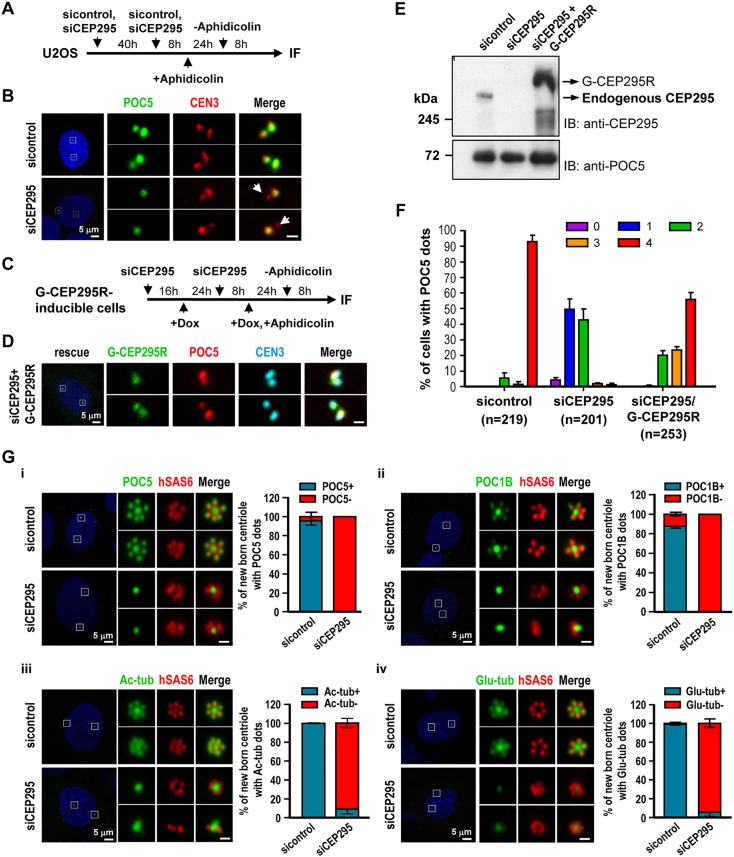
**The G2 centrioles in CEP295-depleted cells fail to recruit POC5 or POC1B and do not show post-translational modification of centriolar microtubules.** (A,B) U2OS cells were treated with sicontrol or siCEP295 as shown in (A) and analyzed by immunofluorescence microscopy (B). Arrows in B indicate inhibited targeting of POC5 to nascent centrioles. (C–F) Rescue experiment. G-CEP295R-inducible cells were treated as described in (C) and analyzed by confocal fluorescence microscopy (D) or immunoblot analysis (E). (F) Histograms illustrating the percentages of cells exhibiting POC5 signals. Error bars represent the mean±s.e.m. from four independent experiments. The total number *n* as indicated. (G) PLK4–myc-inducible cells were treated with siCEP295 as described in Fig. 1C, analyzed by immunofluorescence staining with antibodies against: (i) POC5 and hSAS6; (ii) POC1B and hSAS6; (iii) hSAS6 and Ac-tub, and (iv) hSAS6 and Glu-tub, and expression in new-born centrioles quantified. Error bars represent the mean±s.e.m. *n*=3 independent experiments each scoring 100 cells. Scale bars: 0.5 μm in the enlarged figures.

Our groups (Lin et al., 2013b; Tang et al., 2009) and others (Comartin et al., 2013; Kohlmaier et al., 2009; Schmidt et al., 2009) previously showed that excess CPAP or CEP120 could induce the production of overly long centrioles (>0.5 μm). Conversely, depletion or loss of CEP295 did not appear to affect the initial assembly of cartwheel (Izquierdo et al., 2014) (Fig. 3G), but it did lead to the production of shortened centrioles (Fig. 1). To examine whether CEP295 is required for the process of centriole elongation, we treated CPAP- or CEP120-inducible cells with sicontrol or siCEP295 (Fig. S2). Our results showed that depletion of CEP295 substantially inhibited CPAP-induced centriole elongation (Fig. S2A,B). Intriguingly, nearly 44.0% of CEP295-depleted cells presented with a single mature mother centriole that was positive for CEP164 (a distal appendage marker; Graser et al., 2007) (Fig. S2Bii), whereas the remaining cells contained either two short centrioles (∼47.9%; Fig. S2Biii) or a single long CEP164-labeled centriole (∼0.6%; Fig. S2Biv). This suggests that CEP295 depletion most likely affects CPAP-induced elongation of both mother and new-born centrioles. Similar effects were observed in the CEP120-inducible cells treated with siCEP295 (Fig. S2C,D). Taken together, these results indicate that CEP295 is required for both CPAP- and CEP120-mediated centriole elongation.

### CEP295 is required for the construction of full-length centrioles and the post-translational modification of centriolar microtubules

Recent work showed that POC5 is recruited to centrioles during G2/M and is essential for building the distal portions of centrioles (Azimzadeh et al., 2009). Thus, we examined whether CEP295 depletion affected the localization of POC5 to the distal half centrioles. U2OS cells were treated using the protocol as described in Fig. 3A. Our results showed that depletion of CEP295 substantially inhibited the targeting of POC5 to nascent centrioles in G2-phase U2OS cells (Fig. 3B, arrow). Moreover, these effects were rescued by exogenous expression of a siRNA-resistant G-CEP295R construct (Fig. 3C-3F). A similar phenotype with loss of POC5 was also observed in siCEP295-treated PLK4-inducible cells (Fig. 3Gi). Interestingly, CEP295 depletion also suppressed the recruitment of POC1B, a possible later-born centriolar protein that might control centriole length (Keller et al., 2009), to the G2-nascent centrioles of PLK4-inducible cells (Fig. 3Gii). The relative intensities of POC5 and POC1B at the new-born centrioles in siCEP295-treated PLK4-inducible cells are shown in Fig. S3A and B, respectively. Furthermore, the post-translational modification of centriolar microtubules (e.g. acetylation and glutamylation), which is believed to stabilize centrioles, is known to occur during centriole assembly (Bobinnec et al., 1998; Janke, 2014; Piperno et al., 1987). We found that CEP295 depletion substantially suppressed the acetylation (Fig. 3Giii) and glutamylation (Fig. 3Giv) of microtubules in the G2 centrioles of PLK4-induced cells. Collectively, our results suggest that CEP295 is required for stabilizing centriolar microtubules and the recruitment of POC5 and/or POC1B to distal half centrioles.

### Characterizing the functional domains of CEP295: microtubule interaction and centrosome targeting

**Fig. 4.**
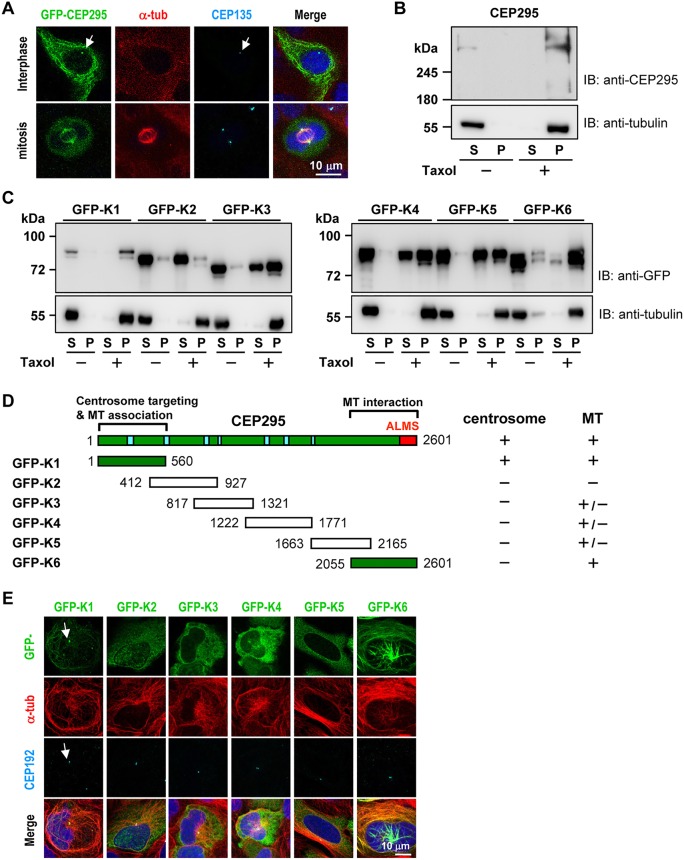
**Mapping the functional domains of CEP295.** (A) CEP295 is associated with interphase and mitotic microtubules. HEK 293T cells were transiently transfected with a vector encoding full-length GFP–CEP295 and analyzed by confocal fluorescence microscopy. Arrow indicates exogenously expressed GFP–CEP295 colocalized with CEP135 at the centrosomes. (B) Endogenous CEP295 co-sediments with microtubules in the presence of Taxol. HEK 293T cells were lysed in lysis buffer in the presence or absence of Taxol. The cell lysates were centrifuged, separated into supernatant (S) and pellet (P) fractions, and analyzed by immunoblotting. (C,D) Co-sedimentation assays (C) with six different fragments of CEP295, GFP−K1 to GFP–K6 (D), were used to map the regions of CEP295 that interact with microtubules. (D) Summary of the functional domains of CEP295: the centrosome-targeting region (centrosome), the microtubule-interacting/associating regions (MT), the ALMS motif (red), and the coil-coiled regions (blue). (E) HEK 293T cells were transfected with vectors encoding various GFP-tagged CEP295 truncation mutants. At 24 h post-transfection, the cells were fixed and stained for α-tubulin and CEP192 (a centrosome marker). Arrow indicates colocalization of N-terminal fragment, GFP–K1, with centrosome marker CEP192.

Our immunogold results showing that CEP295 is located at the centriolar walls (Fig. 2F) led us to speculate that CEP295 might associate with microtubules. To test this possibility, we transfected GFP-tagged full-length CEP295 into HEK 293T cells and performed immunofluorescence microscopy using the indicated antibodies. As shown in Fig. 4, exogenously expressed GFP–CEP295 colocalized with CEP135 at the centrosomes (Fig. 4A, arrow). A substantial portion of the highly expressed GFP–CEP295 was strongly associated with not only interphase microtubules (in 46/67 transfected cells, 68.6%), but also mitotic spindles (15/15, 100%) (Fig. 4A). A microtubule co-sedimentation assay demonstrated that endogenous CEP295 co-sedimented with microtubules *in vivo* in the presence of Taxol (Fig. 4B). To determine the possible microtubule-associating region of CEP295, we transfected HEK 293T cells with various GFP-tagged CEP295 fragments (Fig. 4D) and conducted the microtubule co-sedimentation analyses. Unexpectedly, we did not find evidence of a single specific microtubule-associating region in CEP295; instead, we found two such regions. Both N-terminal (GFP–K1, residues 1–560) and C-terminal (GFP–K6, residues 2055–2601) fragments of CEP295 were efficiently co-precipitated with microtubules and detected in the pellet fractions, whereas the middle regions (K3, K4 and K5) showed only partial associations with microtubules and were found in both supernatant and pellet fractions in the presence of Taxol (Fig. 4C). Consistent with these findings, immunofluorescence analyses revealed that GFP–K1 (50/119, 42%) and GFP–K6 (125/130, 96%), but not the middle fragments, colocalized with microtubules (Fig. 4E). These results suggest that the major microtubule-associating regions of CEP295 are located in the N-terminal K1 fragment and the C-terminal K6 fragment (Fig. 4D). Furthermore, in agreement with Knorz's report (2010), we also mapped the centrosome-targeting region of CEP295 to the N-terminal region, which colocalized with the centrosome marker, CEP192 (Fig. 4E, arrow).

To more precisely map which regions of K1 and K6 could directly bind to microtubules, we subdivided K1 into the K1N and K1C sub-fragments, and K6 into the K6N and K6C sub-fragments (Fig. S4A). We generated GST-fused constructs, affinity purified the GST–recombinant proteins, and performed microtubule sedimentation assays as previously described (Hsu et al., 2008). Of the tested fragments, only GST–K6C bound to and co-sedimented with polymerized microtubules in the pellet fraction (Fig. S4Biii). In agreement with this, GFP-tagged K6C also showed a strong association with microtubules in transfected cells (Fig. S4C). Unexpectedly, GST–K1N (Fig. S4Bi), GST–K1C (Fig. S4Bi), and GST–K6N (Fig. S4B-ii) were prone to auto-precipitating in tubulin-free solution, so we were unable to determine whether their microtubule interactions were direct or indirect. Nonetheless, our findings clearly demonstrate that the C-terminal CEP295-K6C fragment (residues 2472–2601) could directly interact with microtubules (Fig. S4A,Biii). Intriguingly, CEP295-K6C harbors a conserved ALMS motif (Collin et al., 2002; Knorz et al., 2010) of unknown function. We thus speculate that the ALMS motifs of CEP295 and other ALMS-containing proteins (e.g. ALMS1 and C10orf90) might mediate an interaction with microtubules. Furthermore, a modified (nt 1–2472) G-CEP295R construct lacking the ALMS motif (G-CEP295R^1–2472^) could not fully rescue the phenotype of short-length centrioles in siCEP295-treated cells (Fig. S1A,C), implying that the ALMS motif is essential for the full activity of CEP295-mediated centriole elongation.

**Fig. 5.**
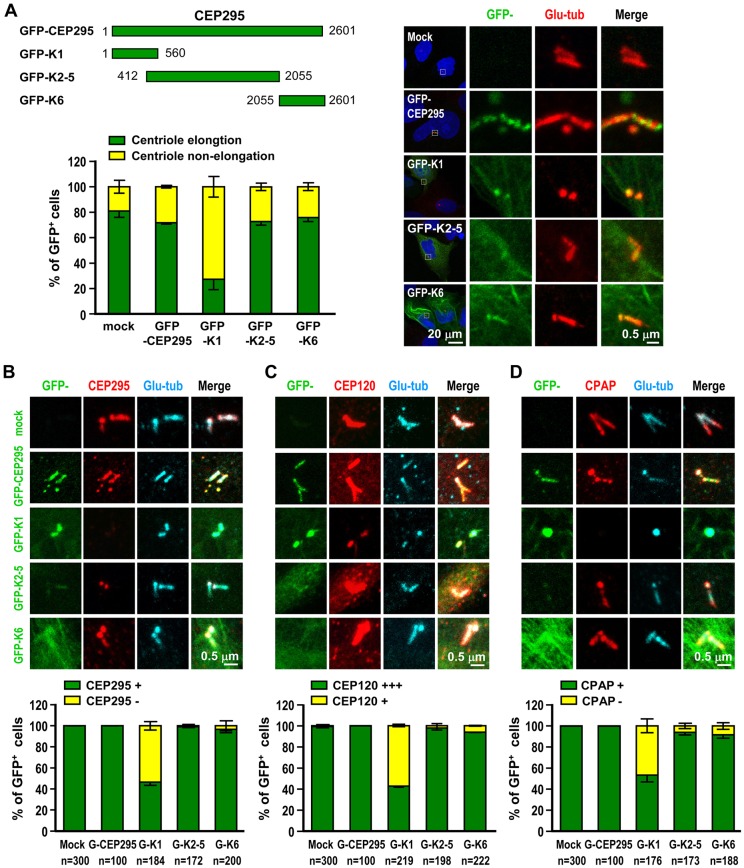
**The N-terminal K1 fragment of CEP295 (GFP–K1) blocks CEP120-induced centriole elongation and suppresses the centriolar localization of CEP295, CPAP and CEP120.** (A) Various GFP–CEP295 truncation mutants were transiently expressed in CEP120–myc-inducible cells. At 24 h after doxycycline induction, the cells were analyzed by confocal fluorescence microscopy, and the percentages of cells with elongated (>0.5 μm) or non-elongated centrioles were calculated. Error bars represent the mean±s.e.m. from three independent experiments (*n*=100 cells/experiment). (B–D) GFP–K1 suppresses the centriolar localization of CEP295, CEP120 and CPAP. Cells treated as described above were fixed and stained with antibodies against CEP295 (B), CEP120 (C), CPAP (D) and polyglutamylated-tubulin (Glu-tub; A–D), and percentages of GFP-positive cells quantified. Error bars represent the mean±s.e.m. from three independent experiments. The total sample size (*n*) as indicated.

To examine whether the microtubule interaction or the centrosomal localization regions of CEP295 are essential for centriole elongation, we transiently transfected vectors encoding full-length GFP–CEP295, GFP–K1, GFP–K2–5, or GFP–K6 into CEP120-inducible cells. We observed that the CEP120-induced formation of overly long centrioles was substantially inhibited in cells expressing GFP–K1, but not GFP–K2–5 or GFP–K6 (Fig. 5A). Interestingly, excess GFP–K1 also substantially suppressed the recruitment of endogenous CEP295 (Fig. 5B), CEP120 (Fig. 5C) and CPAP (Fig. 5D) to centrioles. Based on these findings, we conclude that the N-terminus of CEP295 (K1 fragment) is essential for its centriole-elongating activity. We further speculate that excess K1 might exert a dominant-negative effect by competing with endogenous CEP295 for proper localization and microtubule association.

### CEP295 overexpression induces overly long microtubule-based filaments with low efficiency

**Fig. 6.**
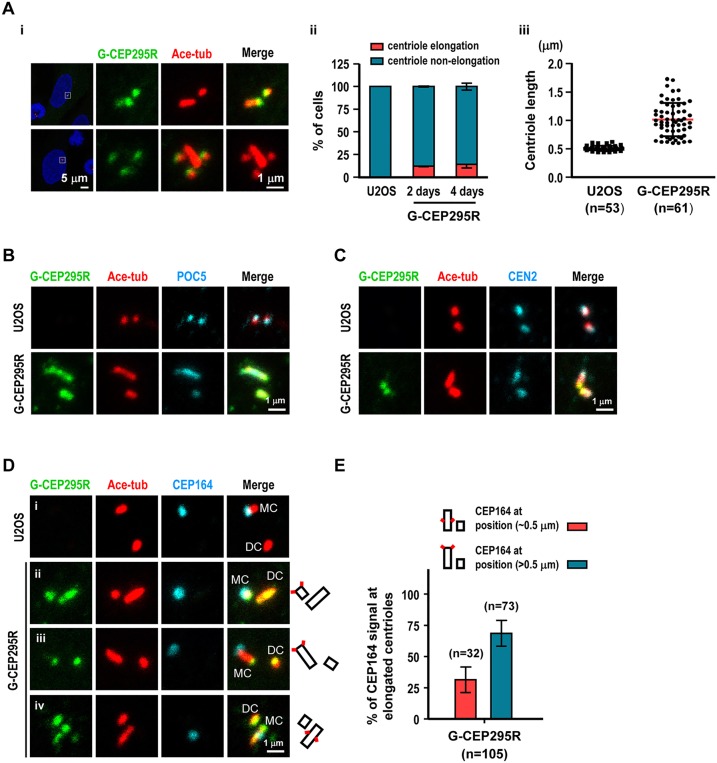
**CEP295 overexpression induces extra-long microtubule-based filaments with low efficiency.** (A) G-CEP295R-inducible cells were treated with doxycycline for 2 days (i-iii) or 4 days (ii), and analyzed by confocal fluorescence microscopy using indicated antibodies. Percentages of cells with elongated centrioles and centriole length were quantified. Centriole length labeled by acetylated tubulin (Ace-tub) >0.5 µm was counted as elongated centriole. Error bars in ii represent the mean±s.e.m. from three independent experiments (*n*=100 cells/experiment). Error bars in iii show the mean±s.d. (B–D) U2OS and G-CEP295R-inducible cells were immunostained with antibodies against Ace-tub and other centriolar proteins including POC5 (B), centrin2 (CEN2; C), and CEP164 (a mother distal appendage marker; D). Schematics on right of D indicate centrioles (black rectangles) and positions of CEP164 (red squares). MC, mother centriole; DC, daughter centriole. (E) The quantitation of percentage of CEP164 signal at elongated centrioles in G-CEP295R-inducible cells. Error bars represent the mean±s.e.m. from two independent experiments. The total number *n* as indicated.

We previously showed that overexpression of CPAP or CEP120 could produce overly long microtubule-based filaments with high efficiency (>50% of transfected cells, Lin et al., 2013b). We next examined whether overexpression of CEP295 could also produce a similar phenotype. The G-CEP295R-inducible cells were treated with or without doxycycline and analyzed by confocal fluorescence microscopy (Fig. 6Ai). Our results showed that less than 18% (Fig. 6Aii) of the G-CEP295R-inducible cells could produce overly long filaments (>0.5 µm; Fig. 6Aiii) that stained positive for Ac-tub, POC5 (Fig. 6B), and centrin (CEN2; Fig. 6C), implying that excess CEP295 alone has a weak activity to produce overly long centriole-like filaments. A similar observation was also found in other G-CEP295R-inducible cell lines (data not shown). Interestingly, a very recent report has shown that Ana1, the *Drosophila* homologue of CEP295, promotes centriole elongation in fly cells indicating that this function is likely conserved (Saurya et al., 2016).

Further immunofluorescence analysis revealed that these long filaments seem to be extending from both CEP164-negative daughter centrioles (Fig. 6Dii) and CEP164-positive mother centrioles (Fig. 6Diii,Div). We found that the long filaments extending from the CEP164-negative daughter centrioles (Fig. 6Dii) are similar to those found in cells overexpression of CPAP (Fig. S2Bi) (Tang et al., 2009) or CEP120 (Fig. S2Di) (Lin et al., 2013b). Surprisingly, a large portion of CEP164-positive centrioles (73/105, 70%) exhibit an unusual position of CEP164 (Fig. 6Diii), which appear to be located at the very distal end (>0.5 µm) of these overly long filaments, whereas the remaining CEP164-positive centrioles (32/105, 30%) show a normal position of the CEP164 signal (∼0.5 µm) on these elongated filaments (Fig. 6Div,E). In contrast, nearly all overly long filaments induced by CPAP or CEP120 overexpression have a normal position (∼0.5 µm) of CEP164 signal on the elongated centrioles (Fig. S2Bi,Di) (Tang et al., 2009; Lin et al., 2013b). Thus, unlike CPAP and CEP120, CEP295 might have an uncharacterized role for the later process of centriole elongation.

### Both CPAP and CEP135 are required for the recruitment of CEP295 to centrioles

**Fig. 7.**
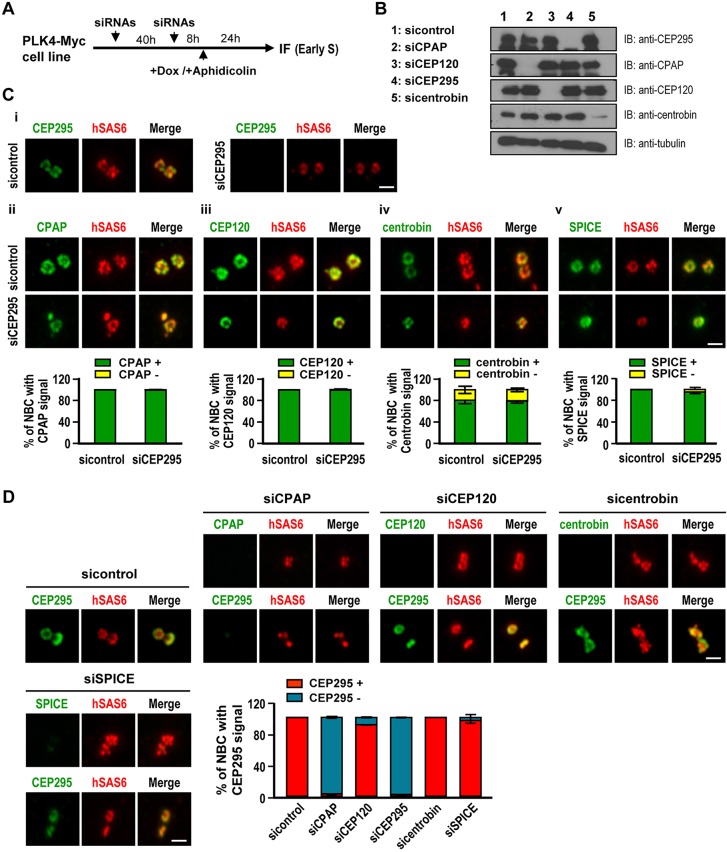
**CEP295 acts downstream of CPAP.** (A) Schematic of the protocol used to analyze the recruitment of centriolar proteins in siRNA-treated PLK4–myc-inducible cells at early S stage. (B) Immunoblot analysis of cell lysates from siRNA-treated cells. (C) Depletion of CEP295 (i-v) did not affect the localizations of CPAP (ii), CEP120 (iii), centrobin (iv) or SPICE (v) to new-born centrioles (NBC). PLK4–myc-inducible cells were treated with sicontrol or siCEP295 as described in A, analyzed by immunofluorescence microscopy, and the results quantified. (D) Depletion of CPAP interferes with the targeting of CEP295 to new-born centrioles (NBC). PLK4–myc-inducible cells were treated with siRNAs against CPAP, CEP120, centrobin or SPICE as described in (A) and analyzed by immunofluorescence microscopy. Scale bars: 0.5 μm. Error bars in C,D represent the mean±s.e.m. *n*=3 independent experiments each scoring 100 cells.

**Fig. 8.**
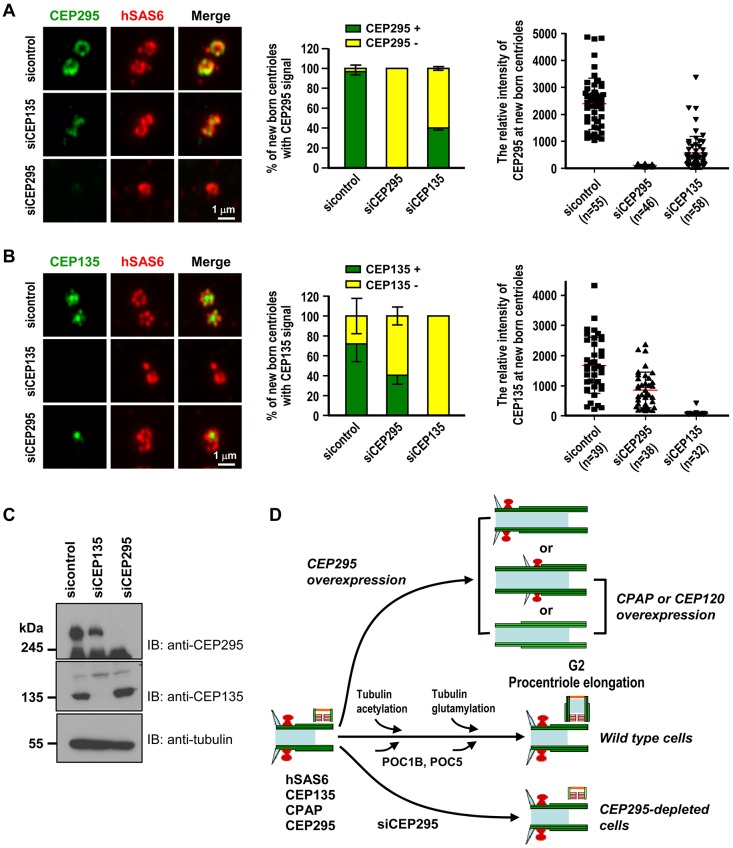
**The localization of CEP295 and CEP135 on new-born centrioles is mutually affected.** (A–C) PLK4–myc-inducible cells were treated with siCEP295 or siCEP135 as described in Fig. 7A and analyzed by confocal immunofluorescence microscopy (A,B) and immunoblotting (C) using indicated antibodies. Percentages of new-born centrioles expressing CEP135 or CEP295 and the relative intensities of CEP135 or CEP295 expression were quantified. Error bars represent the mean±s.e.m. *n*=3 independent experiments each scoring 100 cells or mean±s.d. from indicated numbers (*n*). (D) Model showing how CEP295 is proposed to function in the centriole biogenesis of vertebrate cells. Cyan triangles indicate distal appendages; red mushroom shapes indicate subdistal appendages; brown fence shapes indicate cartwheel structure; orange line indicates the distal end of procentrioles; light blue rectangles indicates the internal lumen of centrioles. For simplicity, we did not include CEP63, CEP152, PLK4 or STIL, which were previously reported to be essential for the early initiation of centriole biogenesis. For detail, please see Discussion.

Recently, several centriolar proteins (CPAP, CEP120, SPICE, CEP135 and centrobin) were reported to play essential roles in centriole duplication and elongation (Comartin et al., 2013; Gudi et al., 2011; Kohlmaier et al., 2009; Lin et al., 2013a,b; Schmidt et al., 2009; Tang et al., 2009). Here, we examined their hierarchical order with respect to CEP295 during the process of centriole elongation. We performed siRNA-mediated depletion of these proteins in U2OS-based PLK4-inducible cells by using the protocol as described in Fig. 7A, and examined by western blotting (Fig. 7B) and the centriolar localization of CEP295 by immunofluorescence microscopy (Fig. 7C,D). These cells were used because the centriolar localizations of the target proteins could be clearly visualized on the rosette structures (which represent the amplified new-born centrioles surrounding the preexisting centrioles) during early S phase upon PLK4 induction (Kleylein-Sohn et al., 2007). As shown in Fig. 7C, depletion of CEP295 did not significantly alter the recruitments of CPAP, CEP120, centrobin, or SPICE to the new-born centrioles in this system. However, the rosettes seemed smaller than those in control cells, possibly reflecting the shortening centrioles observed in CEP295-depleted cells (Fig. 1F). Importantly, we found that depletion of CPAP, but not CEP120, centrobin or SPICE, substantially blocked the targeting of CEP295 to centrioles (Fig. 7D). Furthermore, our results showed that depletion of CEP135 (Fig. 8A,C) perturbs the recruitment of CEP295 to the centrioles (Fig. 8A) and CEP295 depletion (Fig. 8B,C) also perturbs CEP135 targeting to the centrioles (Fig. 8B). A similar finding has independently been reported by Fu et al. (2016). Unexpectedly, our co-IP experiments did not reveal a strong association between CPAP and CEP295 (data not shown). In contrast, it was reported that CEP135 could directly bind to CEP295 and CEP135 seems to load slightly ahead of CEP295 in interphase centrosomes (Fu et al., 2016). Taken together, our results strongly support a model that CPAP and CEP135 might serve as upstream effectors that are required for the recruitment of CEP295 to procentrioles, whereas CEP295 recruitment is possibly mediated by a direct interaction with CEP135.

## DISCUSSION

We and others have previously shown that CEP135 is required for centriole duplication and that depletion of CEP135 significantly reduced centriole numbers in human cells (Kleylein-Sohn et al., 2007; Lin et al., 2013a). We further demonstrated that CEP135 binds to hSAS6 and CPAP, and is required for centriole assembly (Lin et al., 2013a). Interestingly, CEP295 was reported to be directly interacted with CEP135 and CEP152, and such an interaction is essential for centriole-to-centrosome conversion (Fu et al., 2016), yet the role of CEP295 in centriole assembly is not clear.

We herein show that CEP295 directly interacts with microtubules through its ALMS-containing domain, K6C (Fig. S4A), localizes to the centriolar microtubule wall at the proximal ends of centrioles (Fig. 2F), and is required for the generation of the distal half centrioles (Fig. 3). Our data and that from other groups collectively suggest a possible role for CEP295 in the centriole assembly of vertebrate cells. In this proposed model (Fig. 8D), centriole assembly starts during late G1/early S phase after PLK4–STIL activation, which initiates the assembly of the cartwheel with hSAS6 at the central hub (Arquint et al., 2012; Dzhindzhev et al., 2014; Kleylein-Sohn et al., 2007; Kratz et al., 2015; Moyer et al., 2015; Ohta et al., 2014; Strnad et al., 2007; Tang et al., 2011; Vulprecht et al., 2012). CEP135 directly binds to hSAS6 and CPAP, linking the cartwheel to the outer microtubules (Lin et al., 2013a). CPAP then cooperates with CEP120 to promote the assembly of nine triplet microtubules and the extension of procentrioles during S/G2 phase (Comartin et al., 2013; Kohlmaier et al., 2009; Lin et al., 2013b; Schmidt et al., 2009; Tang et al., 2009). During early S phase, CEP295 is possibly recruited in a CPAP- (Fig. 7D) and CEP135-dependent (Fig. 8A) manner to the proximal end of procentrioles at the centriolar microtubule wall (Fig. 2F), possibly through direct interaction with CEP135 (Fu et al., 2016). Our results showed that CPAP and CEP135 might act as upstream effectors of CEP295, as loss of either CPAP (Fig. 7D) or CEP135 (Fig. 8A) (Fu et al., 2016) seem to suppress the recruitment of CEP295 to centrioles. Depletion of CEP295 blocks the incorporation of POC5 and POC1B into the distal portion of centrioles and suppresses the post-translational modification of centriolar microtubules (Fig. 3G). Our study thus uncovers a new role for CEP295 during centriole elongation.

Interestingly, CEP295/Ana1 was also reported to be required for centriole-to-centrosome conversion during mitotic progression in flies and humans (Fu et al., 2016; Izquierdo et al., 2014). It seems that CEP295 might play two distinct roles during cell cycle progression. In interphase (early S phase), CEP295 is first recruited to the proximal end of procentrioles in a CPAP- and CEP135-dependent manner. The successful loading of CEP295 to the procentrioles is required for building the distal half of centrioles in late interphase (Fig. 3G). While in mitosis, CEP295/Ana1 could directly recruit CEP152 or its *Drosophila* homolog, Asl, to new centrioles for the conversion of centrioles to centrosomes (Fu et al., 2016). Thus, the architectural network of CPAP–CEP135–CEP295 is essential for building distal half centrioles in interphase, whereas the CEP135–CEP295–CEP152 network is essential for centriole-to-centrosome conversion in mitosis. Finally, the overly long centriole-like filaments induced by overexpression of CEP295, or CPAP and/or CEP120 exhibit both common and different patterns (Fig. 8D), implying that CEP295 has a unique role for the later process of centriole elongation. The underlined mechanism is currently under investigation.

## MATERIALS AND METHODS

### Plasmids and antibodies

The cDNA encoding full-length CEP295, which was obtained from Dr Thomas Hearn (Knorz et al., 2010), was subcloned into pEGFP-C1 (BD Biosciences Clontech). To generate the siRNA-resistant CEP295 construct, the siRNA-targeted nucleotides within wild-type CEP295 (5′-G ACT GTT AGT GAA ATT GAG AGT AAA) were partially replaced without changing the amino acid sequence (to 5′- G ACT GTT AGC GAG ATA GAA TCT AAA) using a QuikChange kit (Stratagene). To construct the various truncated mutants of CEP295, we generated cDNAs encoding various portions of CEP295 and fused them in-frame to pEGFP-C1 or pGEX4T-1.

To produce a rabbit polyclonal antibody against CEP295, we generated a cDNA encoding amino acids 2092–2430 of CEP295 and inserted it in-frame into the GST cassette of pGEX4T-1. The rabbit polyclonal antibody against CEP295 was raised using recombinant GST–CEP295 (residues 2092–2430) and affinity purified using CEP295 (2092–2430)–His. The antibodies against hSAS6 (1:500 dilution, polyclonal Ab), CPAP (1:1000 dilution), CEP120 (1:1000 dilution), centrobin (1:1000 dilution), CEP135 (1:1000 dilution), and centrin 2 (1:1000 dilution) were as previously described (Tang et al., 2009; Lin et al., 2013a,b). The other commercially available antibodies used in this study included hSAS6 (H00163786, Abnova; 1:200 dilution; monoclonal Ab), STIL (A302-442, Bethyl; 1:200 dilution), centrobin (ab70448, Abcam; 1:1000 dilution; monoclonal Ab), CP110 (12780-1-1p, Proteintech; 1:500 dilution), CEP164 (NBP1-81445, Novus Biologicals; 1:500 dilution), POC5 (A303-341, Bethyl; 1:500 dilution), POC1B (PA5-24495, Thermo Fisher; 1:500 dilution), SPICE (A303-272, Bethyl; 1:500 dilution), centrin3 (H00001070-M01, Abnova; 1:1000 dilution), α-tubulin (T9026, Sigma-Aldrich; 1:100 dilution), acetylated tubulin (T6793, Sigma-Aldrich; 1:1000 dilution) and polyglutamated tubulin (AG-20B-0020, AdipoGen; 1:100 dilution).

### Cell culture, transfection, and synchronization

U2OS and HEK 293 T cells were maintained in Dulbecco's modified Eagle's medium supplemented with 10% fetal calf serum. The U2OS-based doxycycline-inducible CPAP–myc and CEP120–myc cell lines were as described previously (Tang et al., 2009; Lin et al., 2013b). To generate the U2OS-based PLK4–myc inducible cell line or GFP–CEP295R RNAi-resistant inducible line, cDNAs encoding full-length PLK4 or CEP295 siRNA-targeted nucleotides were subcloned into pCDNA4/TO/Myc-His-A and pCDNA4/TO/GFP-Myc-His-A (modified from pCDNA4/TO/Myc-His-A; Invitrogen), respectively. The constructs were transfected into U2OS-T-Rex cells stably expressing the Tet repressor TetR (Tang et al., 2009), using Lipofectamine 2000 (Invitrogen) according to the manufacturer's protocol. The expression of the CPAP–myc, CEP120–myc, PLK4–myc, or GFP–CEP295R RNAi-resistant proteins were induced with 1 μg/ml doxycycline in the culture medium. For synchronization experiments, cells were arrested at early S phase by incubation with aphidicolin (2 μg/ml) for 24 h (Tang et al., 2011), and then released in fresh medium for another 8-15 h (to enrich for G2-phase cells). All cell lines were tested and shown to be without mycoplasma contamination.

### siRNA analysis

The siRNAs used to knock down CPAP, CEP135 and CEP120 were as described previously (Tang et al., 2011; Lin et al., 2013a,b), and those against CEP295, centrobin and SPICE were as follows: siCEP295#1, 5′-GACUGUUAGUGAAAUUGAGAGUAAA; siCEP295#2, 5′-CCAUUUAACUGAACCUUCUUCAUUU; siCEP295#3, 5′-CCAGGUGCCUGUUGCUGACUCUGAA; sicentrobin, 5′-CGAAGCUUGUCAGTCGGAUUGGAAA; siSPICE, 5′-UCUAAACUCUCAGUCUAACACGAAU.

All siRNAs and a non-targeting siRNA control were purchased from Invitrogen. Transfections were performed using Lipofectamine RNAiMAX (Invitrogen) according to the manufacturer's protocol. Although all CEP295 siRNAs work well, siCEP295#1 was used throughout all experiments.

### Immunofluorescence confocal microscopy, super-resolution microscopy and electron microscopy

Immunofluorescence confocal microscopy was performed as previously described (Lin et al., 2013a). Briefly, cells on coverslips were cold treated for 1 h at 4°C and then fixed in methanol at –20°C for 10 min. The cells were blocked with normal goat serum and incubated with primary antibodies. After being washed, the cells were incubated with secondary antibodies conjugated with Alexa Fluor 488 (cat. nos A11034; A11001), Alexa Fluor 568 (cat. nos A11036; A11030) or Alexa Fluor 647 (cat. nos A21245; A21236) (Invitrogen; 1:500 dilution). DNA was counterstained with DAPI. The samples were mounted in Vectashield mounting media (Vector Laboratories), and confocal images were taken using an LSM 780 system or an LSM880 Airyscan system (Carl Zeiss) with a Plan Apochromat 100×/1.4 NA oil-immersion objective. Super-resolution images were acquired using a Zeiss ELYRA system equipped with a 63× Plan-Apochromatic 1.4NA and ZEN software (Carl Zeiss).

Immunogold labeling was performed using a modification of the method described by Fry et al. (1998). Cells grown on Aclar embedding film (Electron Microscopy Sciences) were fixed with 3% paraformaldehyde/2% sucrose in PBS for 10 min, permeabilized with 0.5% Triton X-100/PBS for 2 min, and blocked with 1% BSA/PBS for 30 min. The cells were labeled with anti-CEP295 (1:200) overnight at 4°C, and then with goat anti-rabbit IgG-Nanogold (1:40; Nanoprobes) for 1 h at room temperature. The labeled cells were fixed with 2.5% glutaraldehyde in PBS for 60 min, washed with distilled water, and silver-enhanced for 4 min using HQ Silver reagent (Nanoprobes). After being thoroughly washed with distilled water, the cells were dehydrated in ethanol and embedded in epoxy resin.

Electron microscopy was performed as previously described (Lin et al., 2013a). Briefly, cells grown on Aclar embedding film were rinsed with 0.1 M cacodylate buffer (pH 7.2) containing 4% sucrose and 0.05% CaCl_2_, and fixed with 2.5% glutaraldehyde/1% tannic acid in cacodylate buffer at room temperature for 30 min. The fixed cells were washed three times with cold washing buffer (0.1 M sodium cacodylate/4% sucrose, pH 7.4), post-fixed in 1% OsO_4_/0.1 M cacodylate buffer at room temperature for 30 min, washed three times with cold ddH_2_O, and then stained with 1% uranyl acetate at room temperature for 1 h. The samples were dehydrated in an ethanol series (50, 70, 90 and 100%) and then infiltrated and embedded in Spur resin. Thin sections (80 nm) were generated, and then stained with 4% uranyl acetate and Reynold's lead citrate for 10 min. The samples were examined with an electron microscope (T FEG-TEM; FEI Tecnai G2 TF20 Super TWIN).

### *In vitro* and *in vivo* microtubule co-sedimentation assays

For the *in vitro* microtubule co-sedimentation assays, purified bovine brain α/β-tubulin (5 μM; Cytoskeleton) and various GST–CEP295 truncated recombinant proteins were separately pre-incubated in RG1 buffer (80 mM PIPES pH 6.8, 1 mM EGTA, 1 mM MgCl2, and 1 mM GTP) containing 15 μM Taxol for 30 min at 37°C. After incubation, each GST–CEP295 recombinant protein was separately mixed with incubated tubulin solution. Each reaction mixture was then loaded onto 25 μl of RG1 buffer containing 50% glycerol and incubated at 37°C for 2.5 h. The reaction mixtures were centrifuged at 300,000 ***g*** for 30 min at 37°C in a TLA-100 ultracentrifuge (Beckman Coulter). Supernatants and pellets were subjected to SDS-PAGE analysis followed by Coomassie Blue staining.

The *in vivo* microtubule co-sedimentation assays were performed as previously described (Lin et al., 2013a). Briefly, HEK 293T cells were transfected with vectors encoding GFP–CEP295 truncated mutants. At 24 h post-transfection, the cells were lysed in BRB80 buffer and centrifuged, and the supernatant was incubated with a solution containing 20 μM Taxol. The reaction mixtures were then layered on a cushion buffer (BRB80 buffer with 40% sucrose and 20 μM Taxol) and centrifuged at 100,000 ***g*** for 15 min at 30°C. Finally, the samples were analyzed by western blotting.

### Statistical analysis

Statistical data were analyzed by using Prism 4 (GraphPad Software, La Jolla, CA). Two-tailed unpaired *t*-test was selected to analyze the difference of the value between two groups. ***P<0.0001 was considered statistically significant difference.
